# Pre-Pregnancy Maternal Exposure to Persistent Organic Pollutants and Gestational Weight Gain: A Prospective Cohort Study

**DOI:** 10.3390/ijerph13090905

**Published:** 2016-09-12

**Authors:** Lindsay M. Jaacks, Dana Boyd Barr, Rajeshwari Sundaram, Jagteshwar Grewal, Cuilin Zhang, Germaine M. Buck Louis

**Affiliations:** 1 Department of Global Health and Population, Harvard T.H. Chan School of Public Health, Harvard University, Boston, MA 02115, USA; 2 Department of Environmental Health, Rollins School of Public Health, Emory University, Atlanta, GA 30322, USA; dbbarr@emory.edu; 3 Biostatistics and Bioinformatics Branch, Division of Intramural Population Health Research, *Eunice Kennedy Shriver* National Institute of Child Health and Human Development, Rockville, MD 20852, USA; sundaramr2@mail.nih.gov; 4 Office of the Director, Division of Intramural Population Health Research, *Eunice Kennedy Shriver* National Institute of Child Health and Human Development, Rockville, MD 20852, USA; grewalja@mail.nih.gov (J.G.); louisg@mail.nih.gov (G.M.B.L.); 5 Epidemiology Branch, Division of Intramural Population Health Research, *Eunice Kennedy Shriver* National Institute of Child Health and Human Development, Rockville, MD 20852, USA; zhangcu@mail.nih.gov

**Keywords:** persistent organic pollutants, organochlorine pesticides, pregnancy, obesity

## Abstract

Persistent organic pollutants (POPs) have been implicated in the development of obesity in non-pregnant adults. However, few studies have explored the association of POPs with gestational weight gain (GWG), an important predictor of future risk of obesity in both the mother and offspring. We estimated the association of maternal pre-pregnancy levels of 63 POPs with GWG. Data are from women (18–40 years; *n* = 218) participating in a prospective cohort study. POPs were assessed using established protocols in pre-pregnancy, non-fasting blood samples. GWG was assessed using three techniques: (1) total GWG (difference between measured pre-pregnancy weight and final self-reported pre-delivery weight); (2) category based on pre-pregnancy body mass index (BMI)-specific Institute of Medicine (IOM) recommendations; and (3) area under the GWG curve (AUC). In an exploratory analysis, effects were estimated separately for women with BMI < 25 kg/m^2^ versus BMI ≥ 25 kg/m^2^. Multivariable polytomous logistic regression and linear regression were used to estimate the association between each chemical or congener and the three GWG outcomes. p,p’-dichlorodiphenyl trichloroethane (p,p’-DDT) was significantly inversely associated with AUC after adjustment for lipids and pre-pregnancy BMI: beta {95% confidence interval (CI)}, −378.03 (−724.02, −32.05). Perfluorooctane sulfonate (PFOS) was significantly positively associated with AUC after adjustment for lipids among women with a BMI < 25 kg/m^2^ {beta (95% CI), 280.29 (13.71, 546.86)}, but not among women with a BMI ≥ 25 kg/m^2^ {beta (95% CI), 56.99 (−328.36, 442.34)}. In summary, pre-pregnancy levels of select POPs, namely, p,p’-DDT and PFOS, were moderately associated with GWG. The association between POPs and weight gain during pregnancy may be more complex than previously thought, and adiposity prior to pregnancy may be an important effect modifier.

## 1. Introduction

Persistent organic pollutants (POPs) are a class of compounds that includes pesticides, electrical insulators, surfactants, solvents and flame retardants, among other industrial chemicals. Many POPs were banned in the United States in the late 1970s, but due to their persistence in the environment and bioaccumulation in the food chain, levels are still detectable in nationally-representative samples [1]. Moreover, several POPs were banned more recently, for example the production and import of certain commercial mixtures of polybrominated diphenyl ethers (PBDEs; penta-BDE, which primarily consists of congeners 85, 99 and 100; and octa-BDE, which primarily consists of congeners 183 and 203) were banned in the United States in 2004. Other POPs are not currently banned in the United States, though manufacturers have voluntarily agreed to phase some of them out, for example perfluorooctanoate (PFOA).

POPs have been implicated in the development of obesity in non-pregnant adults [2]. In animal studies, exposure to coplanar polychlorinated biphenyl (PCB) congener 77 and the organochlorine pesticide, hexachlorobenzene (HCB), have been shown to induce weight gain [3,4]. However, to date, few studies have explored the association of POPs with weight gain in humans over short periods of time, such as weight gain during pregnancy. Excess gestational weight gain (GWG) is associated with increased weight retention and obesity in the mother [5], as well as increased risk of obesity in the offspring [6]. Thus, identifying predictors of excess GWG is an important aspect of addressing the obesity epidemic in the United States and other countries [7].

A study of Canadian women (*n* = 1609) found a significant positive association between first-trimester levels of maternal perfluorooctane sulfonate (PFOS) and GWG: beta {95% confidence interval (CI)}, 0.39 (0.02, 0.75) [8]. In contrast, a study in Greece found that women (*n* = 852) with GWG that exceeded the Institute of Medicine’s (IOM) recommendations based on pre-pregnancy body mass index (BMI) had significantly lower levels of 1,1-dichloro-2,2-bis(p-chlorophenyl) ethylene (p,p’-DDE) and PCBs in the first trimester compared to women with GWG below or meeting the recommendations [9]. Similarly, a study in Sweden (*n* = 170–312) found a significant negative association between GWG and PCB congeners 118, 138, 153, 156 and 180 and organochlorine pesticides, HCB and trans-nonachlor, measured late in pregnancy (Weeks 32–34) [10]. Thus, previous studies conducted in Canada and Europe have been inconclusive and have not evaluated a comprehensive panel of POPs. The objective of this analysis was to explore the association between pre-pregnancy levels of 63 POPs and GWG in a prospective cohort of U.S. women.

## 2. Materials and Methods

### 2.1. Study Sample

Data are from a prospective cohort, the Longitudinal Investigation of Fertility and the Environment (LIFE) Study. Details of the cohort have been published previously [11]. Briefly, the sample was recruited between 2005 and 2007 from 16 counties in Michigan and Texas. Eligibility criteria included: (1) married or in a committed relationship; (2) aged 18–40 years for women and ≥18 years for men; (3) self-reported menstrual cycles within the range of 21–42 days; (4) no hormonal birth control injections in the past 12 months; and (5) English- or Spanish-speaking. Among *n* = 1188 eligible participants, *n* = 501 enrolled in the study and *n* = 347 achieved pregnancy (90% within the first six menstrual cycles of attempting pregnancy), of which *n* = 258 completed monthly pregnancy journals for their pregnancies lasting ≥24 weeks gestation. 

Following a baseline study visit, women were followed daily until a positive pregnancy test and through the first seven post-conception weeks of pregnancy, then monthly until delivery. 

The study was conducted in accordance with the Declaration of Helsinki, and the protocol was approved by the Ethics Committees of the National Institutes of Health (OHRP Assurance FWA #00005897; OMB #0925-0542), the EMMES Corporation (IRB #31411), RTI International (IRB #8949), Texas A&M University (IRB #2004), and Emory University (eIRB #80208).

### 2.2. Exposure Assessment 

Pre-pregnancy, non-fasting blood samples were collected during the baseline study visit, spun down and aliquoted immediately, and the plasma was stored at ≤70 °C. Laboratory assessment was conducted by the Division of Laboratory Sciences in the National Center for Environmental Health at the Centers for Disease Control and Prevention using established protocols [12,13]. POPs assessed (63 total) included: one polybrominated biphenyl (PBB 153); ten PBDEs (congeners 17, 28, 47, 66, 85, 99, 100, 153, 154 and 183); 36 PCBs (congeners 28, 44, 49, 52, 66, 74, 87, 99, 101, 105, 110, 114, 118, 128, 138, 146, 149, 151, 153, 156, 157, 167, 170, 172, 177, 178, 180, 183, 187, 189, 194, 195, 196, 201, 206 and 209); nine organochlorine pesticides {HCB, β-hexachlorocyclohexane (β-HCH), γ-hexachlorocyclohexane (γ-HCH), oxychlordane, trans-nonachlor, p,p’-DDT, o,p’-DDT, p,p’-DDE and mirex}; and seven perfluoroalkyls and polyfluoroalkyls {PFAAs; 2-(*N*-ethyl-perfluorooctane sulfonamido) acetate (Et-PFOSA-AcOH), 2-(*N*-methyl-perfluorooctane sulfonamido) acetate (Me-PFOSA-AcOH), perfluorodecanoate (PFDeA), perfluorononanoate (PFNA), perfluorooctane sulfonamide (PFOSA), PFOS and PFOA}. Descriptive statistics of maternal pre-pregnancy levels of these 63 POPs are provided in Table S1. The limit of detections (LOD) for the analytes were calculated by adding a recovery standard to each sample. To calculate the sample-specific LOD, the instrumental LOD was adjusted for the absolute recovery of this standard and background noise for the sample. The mean LOD across all samples (*n* = 218) for PBB 153 was 0.0026 ng/mL; for PBDE 47 0.0114 ng/mL; for PBDE 99 0.0101 ng/mL; for all other PBDE congeners 0.0026 ng/mL; for PCB 28 0.0083 ng/mL; for PCB 52 0.0040 ng/mL; for all other PCB congeners 0.0025 ng/mL; for all organochlorine pesticides 0.0128 ng/mL; for PFNA, PFOS, and PFOA 0.1 ng/mL; and for all other PFAAs 0.2 ng/mL. We did not substitute concentrations below the limit of detection in order to minimize the effect estimate bias associated with this practice [14]. The correlations of each POP with maternal age, pre-pregnancy BMI and non-fasting serum lipids are provided in Table S2.

An enzymatic summation method was used to quantify serum concentrations of total cholesterol, non-esterified cholesterol, triglycerides and phospholipids [15]. Total lipid was calculated using the Phillips formula [16]. 

### 2.3. Gestational Weight Gain Assessment

Pre-pregnancy weight and height were measured during the baseline visit using an established protocol [17]. Briefly, weight was measured twice using a standard digital Health-O-Meter scale and recorded to the nearest pound. If the two measurements differed by more than one pound, a third measurement was taken and recorded. Height was measured twice using a metal tape measure and recorded to the nearest half-inch. If the two measurements differed by more than a half-inch, a third measurement was taken and recorded. Measurements were averaged for analysis. 

**Figure 1:**
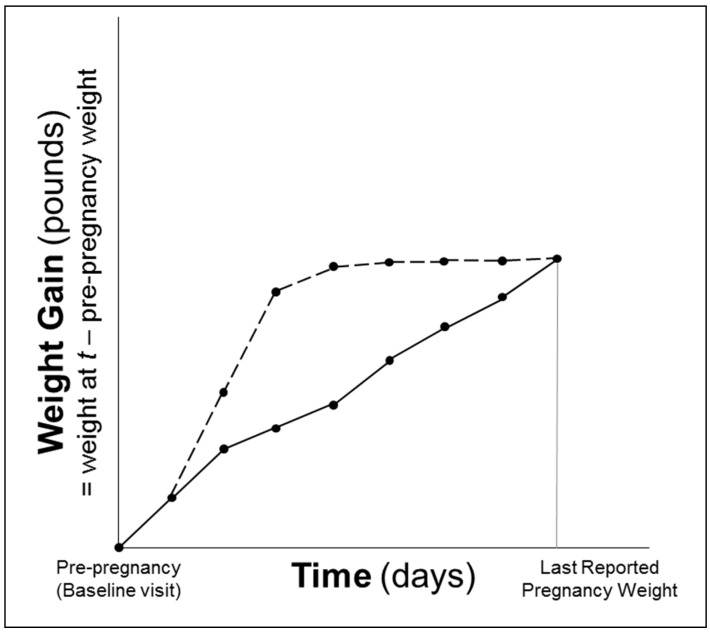
Schematic representation of area under the gestational weight gain curve (AUC). Two lines represent women who gained the same total amount of weight during their pregnancies, but one (dotted line) had a greater AUC, because she gained weight faster earlier in pregnancy.

In monthly pregnancy journals, women were instructed to record their weight. We assessed gestational weight gain using three techniques: (1) total GWG (difference between measured pre-pregnancy weight and final self-reported pre-delivery weight); (2) category based on pre-pregnancy BMI-specific IOM recommendations [18]: (i) gained inadequate weight, (ii) gained adequate weight or (iii) gained excessive weight; and (3) area under the gestational weight gain curve (AUC) [19]. The AUC was calculated by summing the areas of trapezoids formed by successive measures of weight gain in pounds relative to pre-pregnancy weight over the time period in days spanning the baseline (pre-pregnancy) visit to the last self-reported weight before delivery (Figure 1). The AUC is interpreted as the additional pound-days carried by a woman during her pregnancy relative to remaining at her pre-pregnancy weight. For women who self-reported weights during pregnancy that were smaller than their pre-pregnancy weight indicative of weight loss, the values were replaced with their pre-pregnancy weight to avoid negative pound-days [19]. This was the case for *n* = 78 women for the first pregnancy journal (completed at 12 weeks gestation), *n* = 50 for the second (16 weeks gestation), *n* = 29 for the third (20 weeks gestation), *n* = 12 for the fourth (24 weeks gestation), *n* = 6 for the fifth (28 weeks gestation), sixth (32 weeks gestation) and seventh (36 weeks gestation), *n* = 2 for the eighth (40 weeks gestation) and *n* = 1 for the ninth (>40 weeks gestation). On average, women had 7 (range: 2–9) weight values included in the AUC calculation; 99.1% had at least 3 weight values.

### 2.4. Statistical Analysis

All analyses were conducted using SAS software Version 9.4 (SAS Institute, Cary, NC, USA). Markov chain Monte Carlo methods were used to impute missing chemical and lipid data (≤4% missing) using other chemical exposures for the study cohort [20,21]. A total of 10 multiple imputations were computed. POPs and total lipid values were natural log-transformed (x + 1) and rescaled by their standard deviation to aid in the interpretation of results. 

Polytomous logistic regression was used to estimate odds ratios (ORs) and 95% confidence intervals (CIs) for the association between each chemical or congener, specified continuously, and meeting IOM GWG recommendations. Linear regression was used to estimate the association between each chemical or congener, specified continuously, and total GWG and AUC. Covariates were selected for inclusion in multivariable models if they were associated with the exposure, associated with the outcome and not thought to be on the causal pathway [22]. 

Sensitivity analyses were conducted as follows: (1) further adjustment for age; and (2) exclusion of women with gestational diabetes, defined as women who ever reported in monthly pregnancy journals a physician diagnosis of high blood glucose that was identified during pregnancy, not pre-existing (*n* = 27). Universal prenatal glucose screening and testing for GDM is recommended by both the American Diabetes Association [23] and the American College of Obstetricians and Gynecologists [24] with adoption throughout U.S. clinics during the conduct of the LIFE Study. An exploratory analysis estimating the associations between each chemical or congener and total GWG and AUC among women with a pre-pregnancy BMI < 25 kg/m^2^ (*n* = 117) versus women with a BMI ≥ 25 kg/m^2^ (*n* = 101) was also conducted. 

SAS PROC MIANALYZE was used to calculate the average of the 10 complete data estimates from the multiple imputations. *p*-values < 0.05 were considered statistically significant. Given the exploratory nature of this study, we did not adjust for multiple comparisons. 

## 3. Results

Women who did not have a weight recorded within four weeks preceding delivery (*n* = 16; 6.2%) or who were missing a delivery date due to loss to follow-up (*n* = 23; 8.9%) were excluded. One participant had a measured pre-pregnancy weight of 282 pounds (BMI of 50.00 kg/m^2^) and self-reported GWG of 181 pounds and was excluded. One participant was excluded from the AUC analysis because she only had her measured pre-pregnancy weight and her final self-reported pregnancy weight (at delivery); thus, her AUC would assume she carried all of the weight she gained over pregnancy (54 pounds) for the full duration of the pregnancy. Two other participants were excluded from the AUC analysis because they gained over 90 pounds throughout their pregnancy (and over 25 pounds between their pre-pregnancy weight and first pregnancy weight) and, therefore, had AUC values that were over four standard deviations above the mean AUC. The final sample size was therefore *n* = 218 (84.5%) for the analyses of IOM GWG recommendations and total GWG and *n* = 215 (83.3%) for the AUC analysis. 

Only 31% (*n* = 67) of the women met the IOM recommendations for GWG; 41% (*n* = 89) exceeded the recommendations. GWG was not significantly associated with age, race/ethnicity, parity/gravidity, self-reported exercise or serum cotinine (Table 1). Women who gained more than the IOM recommendations had significantly higher pre-pregnancy BMIs and waist circumferences compared to women who gained the recommended amount of weight during pregnancy (*p*-value = 0.0009 and *p*-value = 0.03 for pre-pregnancy BMI and waist circumference, respectively). Pre-pregnancy BMI was inversely associated with AUC (Pearson correlation coefficient = −0.21, *p*-value = 0.002), but not significantly associated with total GWG (Pearson correlation coefficient = −0.08, *p*-value = 0.21). Measured pre-pregnancy waist circumference was inversely associated with both total GWG (Pearson correlation coefficient = −0.30, *p*-value < 0.0001) and AUC (Pearson correlation coefficient = −0.26, *p*-value = 0.0001). Lipids were also inversely associated with total GWG (Pearson correlation coefficient = −0.22, *p*-value = 0.001) and AUC (Pearson correlation coefficient = −0.21, *p*-value = 0.002), but were not associated with whether or not participants met the IOM recommendations for GWG (*p*-value = 0.37).

With regards to meeting the IOM recommendations for GWG, higher pre-pregnancy levels of PCB congeners 177, 183 and 187 were associated with significantly higher odds of gaining less than the IOM recommendations in unadjusted analyses: unadjusted OR (95% CI), 2.29 (1.06, 4.92), 1.97 (1.07, 3.64), 1.72 (1.00, 2.94), respectively. All of these effects were attenuated with adjustment for lipids and pre-pregnancy BMI: adjusted OR (95% CI), 2.20 (0.99, 4.93), 1.82 (0.96, 3.44) and 1.70 (0.96, 3.01), respectively (Table 2). In contrast to the PCB congeners, higher pre-pregnancy levels of γ-HCH were associated with significantly lower odds of gaining less than or more than the IOM recommendations in unadjusted analyses: unadjusted OR (95% CI), 0.60 (0.37, 0.98) and 0.68 (0.47, 0.97), respectively. These results remained significant with further adjustment for lipids and pre-pregnancy BMI: adjusted OR (95% CI), 0.60 (0.37, 0.98) and 0.66 (0.46, 0.97), respectively. In sensitivity analyses, results were consistent with further adjustment for age and after exclusion of women with gestational diabetes (data not shown). However, these results are placed in the context of 99% of samples having γ-HCH concentrations below the LOD (Table S1).

With regards to total GWG, in unadjusted analyses, the only POP statistically significantly associated with total GWG was oxychlordane: unadjusted beta (95% CI), −1.07 (−2.01, −0.13). Trans-nonachlor was marginally non-significantly associated with total GWG: unadjusted beta (95% CI), −0.89 (−1.82, 0.04). After adjustment for lipids and pre-pregnancy BMI, both associations were attenuated: adjusted beta (95% CI), −0.68 (−1.65, 0.30) for oxychlordane and −0.51 (−1.47, 0.44) for trans-nonachlor (Table 2). Both associations also remained non-significant with adjustment for age and after exclusion of women with gestational diabetes (data not shown). However, with further adjustment for age, PBDE congener 99 was significantly associated with total GWG: beta (95% CI), 1.11 (0.05, 2.18). After exclusion of women with gestational diabetes, this result was slightly attenuated: beta (95% CI), 1.07 (−0.02, 2.15). While the association of PBDE congener 154 with total GWG was non-significant in all models of the full sample, after exclusion of women with gestational diabetes, the resulting effect estimate was significant: beta (95% CI), 1.12 (0.02, 2.21).

With regards to area under the gestational weight gain curve, only p,p’-DDT was statistically significantly associated with AUC after adjustment for lipids and pre-pregnancy BMI: adjusted beta (95% CI), −378.03 (−724.02, −32.05) (Table 2). These results were consistent with further adjustment for age and after exclusion of women with gestational diabetes (data not shown). 

In the exploratory analysis of the associations adjusted for lipids and stratified by pre-pregnancy BMI status, no significant associations were observed between POPs and total GWG among women with a pre-pregnancy BMI ≥ 25 kg/m^2^ (Table S3). However, among women with a pre-pregnancy BMI < 25 kg/m^2^, PCB congeners 101 and 151, as well as HCB, were significantly positively associated with total GWG: beta (95% CI), 2.15 (0.28, 4.03) for PCB 101, 8.70 (0.52, 16.87) for PCB 151 and 1.09 (0.06, 2.11) for HCB. These results are placed in the context of 76% of samples having PCB 101 concentrations below the LOD and 99% of samples having PCB 151 concentrations below the LOD (Table S1). For the outcome of area under the gestational weight gain curve, p,p’-DDT was marginally non-significantly negatively associated with AUC among women with a pre-pregnancy BMI < 25 kg/m^2^ {beta (95% CI), −327.73 (−667.66, 12.19)}, but not statistically significantly associated among women with a BMI ≥ 25 kg/m^2^, though the confidence interval was wide {beta (95% CI), −542.53 (−1599.91, 514.84)}. A significant positive association was observed between PFOS and AUC among women with a BMI < 25 kg/m^2^ {beta (95% CI), 280.29 (13.71, 546.86)}, but not among women with a BMI ≥ 25 kg/m^2^ {beta (95% CI), 56.99 (−328.36, 442.34)}. In contrast, a significant negative association was observed between PBDE congener 66 and AUC among women with a BMI ≥ 25 kg/m^2^ {beta (95% CI), −376.04 (−734.05, −18.03)}, but not among women with a BMI < 25 kg/m^2^ {beta (95% CI), −31.72 (−298.28, 234.83)}. This result is placed in the context of 87% of samples having PBDE 66 concentrations below the LOD (Table S1).

## 4. Discussion

This is the first study to prospectively evaluate the association of pre-pregnancy serum levels of POPs with weight gain throughout pregnancy. We hypothesized that high levels of POPs pre-pregnancy would increase risk of gaining excessive weight throughout pregnancy given that POPs have previously been shown to alter cell signaling involved in weight homeostasis, particularly as relates to peroxisome proliferator-activated receptors involved in adipogenesis [25,26]. We found that pre-pregnancy levels of select POPs, namely, p,p’-DDT and PFOS, were associated with GWG. Higher pre-pregnancy levels of p,p’-DDT were associated with carrying fewer pound-days of weight throughout pregnancy, among both normal weight and overweight/obese women. A significant positive association was observed between PFOS and AUC among normal weight women, but not among overweight/obese women. These results suggest that the effects of POPs on weight during pregnancy when fat compartments in both the mom and fetus are changing are more complex than previously thought. The opposite direction of association for p,p’-DDT versus PFOS may indicate that effects are influenced by whether or not the POPs under question are lipophilic. 

One previous study reported that mean serum levels of POPs, including HCB, p,p’-DDE, total PCBs, dioxin-like PCBs and non-dioxin-like PCBs, were significantly lower among women who gained excessive weight during pregnancy [9]. Consistent with this, we observed lower levels of γ-HCH among women who gained excessive weight compared to women who gained adequate weight. In our study, among women who gained adequate weight, the mean (SD) γ-HCH was 1.18 (2.63) pg/mL compared to 0.30 (1.56) pg/mL among women gaining inadequate weight and 0.40 (1.40) pg/mL among women gaining excessive weight. We did not find significant differences across IOM GWG categories for any other POPs. 

PFAAs are surfactants used widely in consumer products, including cookware, food packaging and clothing. Interestingly, we observed a significant positive association between PFOS and area under the gestational weight gain curve among women with a BMI < 25 kg/m^2^, but not among women with a BMI ≥ 25 kg/m^2^. No significant associations were observed for any other PFAAs. This is consistent with the results of one previous study, conducted in Canada, which found that higher maternal PFOS levels in the first trimester were associated with modest increases in GWG among women with pre-pregnancy BMIs < 25 kg/m^2^, but not among women with BMIs ≥ 25 kg/m^2^ [8]. They also did not find an association between PFOA or perfluorohexanesulfanoate (PFHxS) and GWG [8]. 

We found that higher pre-pregnancy levels of p,p’-DDT were associated with carrying fewer pound-days of weight throughout pregnancy. Previous epidemiological studies have only reported on p,p’-DDE, a metabolite/degradate of p,p’-DDT, and have similarly reported that higher levels of p,p’-DDE in either the first or third trimester were associated with lower levels of GWG [9,10]. Animal studies have found that at high levels, select POPs, such as coplanar PCB congener 77 and 2,3,7,8-tetrachlorodibenzo-p-dioxin (TCDD), inhibit adipocyte differentiation [3,27]. 

A key strength of this study was that it was prospective and included a standardized anthropometric assessment for measuring pre-pregnancy BMI along with a comprehensive, state-of-the-art laboratory assessment of POPs exposure. The use of self-reported GWG was a limitation, though women were encouraged to take their pregnancy journals to all obstetrical visits for capture of measured weight and noting any other antenatal test results. Furthermore, studies of self-reported GWG in the United States have found that even when recalled up to one year postpartum, self-reported GWG correctly classifies most women according to IOM recommendations when compared to birth certificate data [28]. Thus, because women in the LIFE study were self-reporting weight monthly throughout pregnancy, the measurement error is likely negligible. Overall, *n* = 3 (1.4%) women lost weight from pre-pregnancy to their last self-reported pregnancy weight. A previous analysis of the Behavioral Risk Factor Surveillance System (BRFSS) in the United States found that 7.5% of pregnant women reported trying to lose weight during their pregnancy and 34.3% reported trying to maintain their pre-pregnancy weight [29]; thus, this observation is not surprising. The small sample size is also a limitation, and null effects should be quantified in other observational studies and meta-analyses. 

We only had a single, pre-pregnancy measure of POPs, but the persistent nature of such compounds given their long half-lives (years) should minimize marked fluctuations. Of the three studies that previously evaluated the association of select POPs with GWG, two measured maternal POPs in the first trimester [8,9] and the third measured maternal POPs later in pregnancy (Weeks 32–34) [10]. A longitudinal study of serum PCB levels from pre-pregnancy through delivery showed that levels declined among women who achieved pregnancy during the peri-conception time window [30]. Thus, the pre-pregnancy measures used in our study likely represent the highest levels experienced by women during the pre-pregnancy/pregnancy period short of new exposures occurring during pregnancy. 

## 5. Conclusions

Pre-pregnancy levels of select POPs, namely, p,p’-DDT and PFOS, were associated with GWG. Cumulative low-level exposures to POPs may be more strongly associated with weight gain over long periods of time rather than short periods of time, such as that occurring during pregnancy. In addition, the role of POPs as potential obesogens needs to be carefully considered in light of lipid compartment dynamics, including both adipose tissue and circulating lipids, which are important determinants of measured levels of POPs. Adiposity in the period leading up to pregnancy may be an important modifier of the effects of POPs on weight gain during pregnancy. Further research is needed to improve our understanding of how changes in POPs exposure throughout gestation influence weight-gain dynamics. 

## Figures and Tables

**Table 1:** Characteristics of women participating in the Longitudinal Investigation of Fertility and the Environment (LIFE) Study who achieved pregnancy lasting ≥24 weeks gestation and submitted a pregnancy journal according to gestational weight gain status (*n* = 218) ^1^.

Maternal Characteristics	IOM GWG Recommendations			*p*-Value ^3^
	Adequate (*n* = 67)	Inadequate (*n* = 62)	Excessive (*n* = 89)	
Pre-pregnancy age (years)	29.4 (3.5)	30.1 (4.0)	29.6 (3.8)	0.59
Pre-pregnancy BMI (kg/m^2^)	24.2 (4.6)	25.9 (7.7)	27.9 (6.0)	0.0009
Pre-pregnancy waist circumference (cm)	84.1 (13.6)	88.3 (18.0)	90.5 (12.9)	0.03
Non-fasting serum lipids (mg/dL) ^2^	604.9 (124.4)	633.8 (121.7)	618.9 (106.9)	0.37
Serum cotinine (ng/mL)	10.0 (37.4)	14.5 (80.5)	13.1 (49.3)	0.90
Gestational weight gain (kg)	13.4 (3.1)	7.1 (4.5)	18.6 (6.7)	<0.0001
Parity and gravidity (%)				
Never pregnant	40.3 (27)	37.1 (23)	42.7 (38)	0.34
Pregnant without live birth	11.9 (8)	4.8 (3)	4.5 (4)	
Pregnant with previous birth	47.8 (32)	58.1 (36)	52.8 (47)	
Gestational diabetes (%)				
Yes	14.9 (10)	21.0 (13)	4.6 (4)	0.009
No	85.1 (57)	79.0 (49)	95.4 (83)	
Race/ethnicity (%)				
Non-Hispanic white	82.1 (55)	82.3 (51)	86.5 (77)	0.69
Other	17.9 (12)	17.7 (11)	13.5 (12)	
Follow regular vigorous exercise program (%)				
Yes	43.3 (29)	50.0 (31)	39.3 (35)	0.43
No	56.7 (38)	50.0 (31)	60.7 (54)	

Values are the mean (SD) or % (n). BMI: body mass index; GWG: gestational weight gain; IOM: Institute of Medicine. ^1^ Gestational weight gain status defined according to the Institute of Medicine recommendations as: For pre-pregnancy BMI < 18.5 kg/m^2^, 25–35 lbs; for pre-pregnancy BMI ≥ 18.5 and < 25 kg/m^2^, 28–40 lbs; for pre-pregnancy BMI ≥ 25 and < 30 kg/m^2^, 15–25 lbs; and for pre-pregnancy BMI ≥ 30 kg/m^2^, 11–20 lbs. ^2^ From the Phillips 1989 equation. ^3^ *p*-value from the chi-square test for categorical variables and analysis of variance for continuous variables.

**Table 2:** Estimated associations between persistent organic pollutants and gestational weight gain.

Chemical or Congener	IOM GWG Recommendations ^1^		Total GWG ^2^ Beta (95% CI)	AUC ^2^ Beta (95% CI)
	Inadequate OR (95% CI)	Excessive OR (95% CI)		
Polybrominated biphenyl (PBB)				
PBB 153	1.11 (0.80, 1.52)	0.79 (0.52, 1.21)	−0.70 (−1.66, 0.26)	−188.62 (−416.34, 39.11)
Polybrominated diphenyl ethers (PBDE)				
PBDE 17	0.90 (0.65, 1.26)	0.84 (0.59, 1.20)	−0.12 (−1.01, 0.77)	−66.63 (−275.77, 142.51)
PBDE 28	0.98 (0.70, 1.38)	0.77 (0.51, 1.17)	−0.13 (−1.15, 0.89)	−170.00 (−410.59, 70.59)
PBDE 47	0.77 (0.51, 1.15)	0.76 (0.53, 1.10)	0.52 (−0.50, 1.55)	−134.47 (−387.02, 118.08)
PBDE 66	0.98 (0.69, 1.38)	0.85 (0.59, 1.21)	−0.60 (−1.54, 0.35)	−149.42 (−369.62, 70.79)
PBDE 85	0.67 (0.40, 1.11)	0.72 (0.49, 1.07)	0.87 (−0.25, 1.99)	−59.61 (−340.03, 220.82)
PBDE 99	0.58 (0.33, 1.04)	0.74 (0.50, 1.08)	1.07 (0.00, 2.14)	−29.29 (−303.56, 244.98)
PBDE 100	0.87 (0.61, 1.25)	0.86 (0.62, 1.20)	0.18 (−0.78, 1.14)	−37.15 (−266.77, 192.46)
PBDE 153	1.01 (0.72, 1.40)	1.00 (0.74, 1.37)	0.05 (−0.83, 0.94)	70.64 (−139.52, 280.79)
PBDE 154	0.78 (0.50, 1.21)	0.77 (0.53, 1.14)	0.71 (−0.33, 1.74)	-89.74 (−358.65, 179.18)
PBDE 183	1.48 (0.92, 2.36)	1.38 (0.87, 2.20)	−0.34 (−1.23, 0.55)	−92.64 (−304.01, 118.73)
Polychlorinated biphenyls (PCB)				
PCB 28	0.27 (0.00, 76.04)	1.34 (0.18, 9.84)	−0.35 (−1.20, 0.50)	−5.94 (−208.80, 196.93)
PCB 44	0.75 (0.02, 33.62)	3.30 (0.17, 64.18)	−0.29 (−1.15, 0.57)	4.63 (−199.72, 208.98)
PCB 49	0.29 (0.01, 8.23)	1.17 (0.55, 2.49)	−0.30 (−1.15, 0.56)	−0.76 (−204.34, 202.83)
PCB 52	0.09 (0.00, 10.68)	1.31 (0.22, 7.65)	−0.29 (−1.14, 0.56)	3.90 (−199.10, 206.90)
PCB 66	1.17 (0.04, 36.10)	2.14 (0.09, 49.19)	−0.34 (−1.19, 0.51)	−4.42 (−207.29, 198.44)
PCB 74	1.17 (0.47, 2.93)	1.33 (0.56, 3.16)	−0.28 (−1.14, 0.58)	−3.36 (−207.44, 200.73)
PCB 87	0.89 (0.56, 1.43)	1.14 (0.78, 1.67)	0.08 (−0.84, 0.99)	63.27 (−155.35, 281.89)
PCB 99	1.14 (0.76, 1.70)	1.13 (0.77, 1.65)	−0.09 (−1.07, 0.90)	0.97 (−234.43, 236.37)
PCB 101	0.76 (0.39, 1.49)	1.05 (0.69, 1.61)	−0.25 (−1.38, 0.88)	16.69 (−255.24, 288.62)
PCB 105	1.14 (0.76, 1.72)	1.09 (0.73, 1.61)	−0.29 (−1.31, 0.72)	−41.23 (−283.11, 200.66)
PCB 110	1.01 (0.70, 1.46)	1.00 (0.72, 1.40)	−0.31 (−1.24, 0.61)	−29.07 (−252.09, 193.94)
PCB 114	0.94 (0.66, 1.34)	0.97 (0.71, 1.33)	0.03 (−0.89, 0.95)	22.50 (−195.91, 240.91)
PCB 118	1.12 (0.74, 1.69)	1.08 (0.73, 1.59)	−0.15 (−1.17, 0.88)	−3.23 (−245.23, 238.77)
PCB 128	0.64 (0.36, 1.15)	0.75 (0.49, 1.16)	0.02 (−1.27, 1.31)	−105.69 (−440.61, 229.23)
PCB 138	1.39 (0.91, 2.13)	1.17 (0.77, 1.78)	−0.25 (−1.32, 0.83)	−42.40 (−297.55, 212.75)
PCB 146	1.21 (0.80, 1.82)	1.05 (0.70, 1.59)	−0.17 (−1.24, 0.90)	−50.26 (−305.14, 204.61)
PCB 149	3.19 (0.46, 21.95)	1.89 (0.28, 12.99)	−2.92 (−7.04, 1.21)	−316.35 (−1359.90, 727.20)
PCB 151	8.82 (0.21, 364)	6.12 (0.15, 252)	−1.84 (−5.51, 1.84)	−518.00 (−1419.83, 383.82)
PCB 153	1.33 (0.87, 2.04)	1.08 (0.71, 1.66)	−0.18 (−1.29, 0.93)	−61.67 (−325.57, 202.24)
PCB 156	1.05 (0.74, 1.47)	0.85 (0.60, 1.22)	0.03 (−0.94, 0.99)	−80.63 (−309.63, 148.37)
PCB 157	0.93 (0.66, 1.32)	0.91 (0.66, 1.27)	−0.05 (−0.98, 0.89)	−49.87 (−272.16, 172.43)
PCB 167	0.96 (0.64, 1.42)	1.12 (0.79, 1.59)	0.49 (−0.50, 1.48)	144.88 (−89.54, 379.30)
PCB 170	1.36 (0.87, 2.13)	1.07 (0.68, 1.67)	−0.09 (−1.26, 1.08)	−92.31 (−370.58, 185.96)
PCB 172	1.13 (0.76, 1.68)	0.88 (0.59, 1.32)	−0.44 (−1.51, 0.64)	−71.72 (−327.26, 183.81)
PCB 177	2.20 (0.99, 4.93)	1.89 (0.86, 4.16)	−0.16 (−1.96, 1.64)	61.23 (−367.63, 490.09)
PCB 178	1.41 (0.90, 2.22)	1.14 (0.73, 1.79)	−0.02 (−1.20, 1.16)	78.32 (−201.73, 358.37)
PCB 180	1.33 (0.86, 2.06)	1.07 (0.69, 1.65)	0.04 (−1.11, 1.19)	−34.92 (−307.94, 238.09)
PCB 183	1.82 (0.96, 3.44)	1.47 (0.79, 2.75)	−0.35 (−1.85, 1.16)	75.20 (−282.92, 433.31)
PCB 187	1.70 (0.96, 3.01)	1.29 (0.73, 2.28)	−0.23 (−1.57, 1.10)	−44.82 (−362.42, 272.78)
PCB 189	1.08 (0.77, 1.50)	1.00 (0.71, 1.39)	−0.18 (−1.11, 0.74)	−41.17 (−261.29, 178.96)
PCB 194	1.14 (0.78, 1.68)	1.05 (0.72, 1.54)	0.46 (−0.57, 1.48)	61.33 (−184.78, 307.44)
PCB 195	1.31 (0.86, 2.00)	1.29 (0.86, 1.94)	0.11 (−0.97, 1.20)	89.48 (−167.30, 346.25)
PCB 196	1.30 (0.84, 2.02)	1.13 (0.74, 1.74)	0.36 (−0.77, 1.49)	84.07 (−185.46, 353.61)
PCB 201	1.12 (0.72, 1.74)	1.03 (0.67, 1.57)	0.40 (−0.74, 1.55)	101.50 (−174.03, 377.04)
PCB 206	1.17 (0.80, 1.72)	0.99 (0.67, 1.46)	0.20 (−0.83, 1.23)	110.49 (−133.68, 354.66)
PCB 209	1.28 (0.90, 1.83)	1.00 (0.70, 1.43)	0.12 (−0.79, 1.04)	86.60 (−130.28, 303.48)
Organochlorine pesticides				
HCB	0.83 (0.56, 1.23)	0.90 (0.64, 1.28)	0.43 (−0.56, 1.43)	−70.65 (−315.78, 174.47)
β-HCH	1.04 (0.66, 1.66)	1.14 (0.77, 1.71)	0.62 (−0.28, 1.53)	−118.52 (−612.00, 374.96)
γ-HCH	**0.60** **(0.37, 0.98)**	**0.66** **(0.46, 0.97)**	0.25 (−0.75, 1.25)	39.43 (−197.41, 276.27)
Oxychlordane	1.29 (0.90, 1.86)	0.91 (0.62, 1.33)	−0.68 (−1.65, 0.30)	−135.40 (−367.24, 96.44)
Trans-nonachlor	1.17 (0.82, 1.66)	0.91 (0.62, 1.34)	−0.51 (−1.47, 0.44)	−50.18 (−278.41, 178.06)
p,p’-DDT	1.09 (0.77, 1.56)	1.07 (0.75, 1.53)	0.51 (−0.37, 1.38)	**−378.03** **(−724.02, −32.05)**
o,p’-DDT	1.06 (0.74, 1.51)	1.06 (0.75, 1.48)	0.63 (−0.30, 1.57)	−177.83 (−497.71, 142.05)
p,p’-DDE	1.13 (0.79, 1.61)	1.06 (0.75, 1.51)	0.22 (−0.73, 1.16)	−78.61 (−334.15, 176.92)
Mirex	0.99 (0.49, 1.98)	1.17 (0.65, 2.09)	0.27 (−1.33, 1.88)	289.39 (−86.94, 665.71)
Perfluoroalkyls and polyfluoroalkyls (PFAAs)				
Et-PFOSA-AcOH	1.04 (0.73, 1.48)	0.87 (0.62, 1.24)	−0.32 (−1.28, 0.65)	−3.69 (−231.46, 224.08)
Me-PFOSA-AcOH	0.97 (0.70, 1.35)	0.82 (0.59, 1.14)	−0.28 (−1.18, 0.62)	−93.23 (−308.78, 122.33)
PFDeA	0.99 (0.69, 1.42)	1.12 (0.81, 1.55)	0.00 (−0.90, 0.90)	50.68 (−165.79, 267.15)
PFNA	0.78 (0.54, 1.13)	0.98 (0.69, 1.38)	−0.03 (−1.00, 0.94)	84.92 (−145.21, 315.06)
PFOSA	0.74 (0.49, 1.11)	0.93 (0.69, 1.26)	0.60 (−0.31, 1.51)	112.99 (−103.87, 329.85)
PFOS	0.70 (0.48, 1.01)	1.01 (0.72, 1.40)	0.26 (−0.66, 1.18)	178.43 (−43.01, 399.87)
PFOA	0.87 (0.61, 1.23)	1.06 (0.76, 1.47)	0.09 (−0.84, 1.02)	0.75 (−220.50, 222.01)

AUC: Area under the curve; BMI: body mass index; CI: confidence interval; GWG: gestational weight gain; IOM: Institute of Medicine; OR: Odds ratio; HCB: Hexachlorobenzene; HCH: Hexachlorocyclohexane; p,p’-DDT: p,p’-Dichlorodiphenyl trichloroethane; o,p’-DDT: o,p’-Dichlorodiphenyl trichloroethane; p,p’-DDE: 1,1-Dichloro-2,2-bis(p-chlorophenyl) ethylene; Et-PFOSA-AcOH: 2-(*N*-ethyl-perfluorooctane sulfonamido) acetate; Me-PFOSA-AcOH: 2-(*N*-methyl-perfluorooctane sulfonamido) acetate; PFDeA: Perfluorodecanoate; PFNA: Perfluorononanoate; PFOS: Perfluorooctane sulfonate; PFOA: Perfluorooctanoate. ^1^ Values are OR (95% CI) estimated from multivariable polytomous regression. Referent: adequate GWG. Adjusted for pre-pregnancy non-fasting serum lipids and BMI. ^2^ Values are beta (95% CI) estimated from multivariable linear regression. Adjusted for pre-pregnancy non-fasting serum lipids and BMI.

## Supplementary Materials

The following are available online at www.mdpi.com/1660-4601/13/9/905/s1: Table S1: Descriptive statistics of pre-pregnancy maternal levels of persistent organic pollutants (ng/mL), Table S2: Correlations between pre-pregnancy persistent organic pollutant levels and maternal age, body mass index (BMI) and serum lipids, Table S3: Estimates from linear regression of the association between persistent organic pollutants and gestational weight gain stratified by pre-pregnancy body mass index (BMI) status.
