# Supplementary material for: Pre-Pregnancy Maternal Exposure to Persistent Organic Pollutants and Gestational Weight Gain: A Prospective Cohort Study
Source: Int J Environ Res Public Health. 2016 Sep 12;13(9):905. doi: 10.3390/ijerph13090905 (PMC5036738; doi:10.3390/ijerph13090905)
Supplement: Supplementary file 1 [file ijerph-13-00905-s001.pdf]

Supplementary Materials: Pre-Pregnancy Maternal Exposure to Persistent Organic Pollutants and Gestational Weight Gain: A Prospective Cohort Study

Lindsay M. Jaacks, Dana Boyd Barr, Rajeshwari Sundaram, Jagteshwar Grewal, Cuilin Zhang and Germaine M. Buck Louis

1. Table S1

Table S1. Descriptive statistics of pre-pregnancy maternal levels of persistent organic pollutants (ng/mL).

Chemical or Congener	% < LOD [a]	Mean (SD)
Polybrominated biphenyl (PBB)		
PBB 153	8.7	0.0151 (0.0293)
Polybrominated diphenyl ethers (PBDE)		
PBDE 17	83.0	0.0014 (0.0036)
PBDE 28	24.3	0.0123 (0.0180)
PBDE 47	0.9	0.2156 (0.3036)
PBDE 66	87.2	0.0012 (0.0025)
PBDE 85	59.6	0.0043 (0.0080)
PBDE 99	24.3	0.0432 (0.0869)
PBDE 100	1.4	0.0515 (0.0891)
PBDE 153	0.5	0.1023 (0.2094)
PBDE 154	59.2	0.0042 (0.0077)
PBDE 183	84.4	0.0018 (0.0022)
Polychlorinated biphenyls (PCB)		
PCB 28	67.4	0.0314 (0.3611)
PCB 44	87.6	0.0025 (0.0179)
PCB 49	95.4	0.0009 (0.0065)
PCB 52	96.3	0.0022 (0.0175)
PCB 66	43.1	0.0066 (0.0436)
PCB 74	0	0.0182 (0.0357)
PCB 87	92.7	0.0007 (0.0013)
PCB 99	1.8	0.0125 (0.0096)
PCB 101	75.7	0.0022 (0.0035)
PCB 105	29.8	0.0044 (0.0037)
PCB 110	89.5	0.0013 (0.0020)
PCB 114	90.4	0.0009 (0.0014)
PCB 118	0.5	0.0203 (0.0148)
PCB 128	97.3	0.0001 (0.0006)
PCB 138	1.4	0.0379 (0.0275)
PCB 146	17.4	0.0058 (0.0050)
PCB 149	96.8	0.0004 (0.0010)
PCB 151	98.6	0.0003 (0.0014)
PCB 153	0	0.0534 (0.0370)
PCB 156	11.5	0.0072 (0.0060)
PCB 157	78.4	0.0015 (0.0018)
PCB 167	78.9	0.0011 (0.0020)
PCB 170	2.3	0.0150 (0.0101)
PCB 172	79.8	0.0013 (0.0017)

PCB 177	60.6	0.0025 (0.0027)
PCB 178	63.3	0.0022 (0.0026)
PCB 180	0	0.0379 (0.0247)
PCB 183	31.7	0.0043 (0.0040)
PCB 187	6.4	0.0123 (0.0100)
PCB 189	98.6	0.0004 (0.0007)
PCB 194	13.3	0.0079 (0.0060)
PCB 195	75.2	0.0014 (0.0018)
PCB 196	9.2	0.0083 (0.0060)
PCB 201	15.6	0.0077 (0.0062)
PCB 206	30.3	0.0041 (0.0033)
PCB 209	81.2	0.0019 (0.0014)
Organochlorine pesticides		
HCB	1.4	0.0503 (0.0228)
β-HCH	59.6	0.0241 (0.1230)
γ-HCH	99.5	0.0006 (0.0019)
Oxychlordane	15.6	0.0360 (0.0297)
Trans-nonachlor	3.2	0.0662 (0.0657)
p,p′-DDT	59.6	0.0158 (0.0270)
o,p′-DDT	99.5	0.0015 (0.0029)
p,p′-DDE	0	0.7925 (1.1370)
Mirex	80.7	0.0117 (0.0243)
Perfluoroalkyls and polyfluoroalkyls (PFAAs)		
Et-PFOSA-AcOH	99.5	0.0317 (0.0564)
Me-PFOSA-AcOH	44.5	0.4276 (0.4478)
PFDeA	23.9	0.4727 (0.4117)
PFNA	2.3	1.3540 (0.7510)
PFOSA	89.5	0.0120 (0.0366)
PFOS	0	14.8120 (11.2803)
PFOA	0.5	3.6580 (1.9728)

LOD: limit of detection; SD: standard deviation; [a] The mean LOD across all samples (n = 218) for PBB 153 was 0.0026 ng/mL; for PBDE 47 was 0.0114 ng/mL; for PBDE 99 was 0.0101 ng/mL; for all other PBDE congeners was 0.0026 ng/mL; for PCB 28 was 0.0083 ng/mL; for PCB 52 was 0.0040 ng/mL; for all other PCB congeners was 0.0025 ng/mL; for all organochlorine pesticides was 0.0128 ng/mL; for PFNA, PFOSA, and PFOA was 0.1 ng/mL; and for all other PFAAs was 0.2 ng/mL.

2. Table S2

Table S2. Correlations between pre-pregnancy persistent organic pollutant levels and maternal age, body mass index (BMI), and serum lipids.

Chemical or Congener	Age	BMI	Serum Lipids
Polybrominated biphenyl (PBB)			
PBB 153	0.10 (0.14)	0.02 (0.80)	0.00 (0.98)
Polybrominated diphenyl ethers (PBDE)			
PBDE 17	0.13 (0.07)	0.03 (0.66)	0.05 (0.50)
PBDE 28	0.07 (0.32)	0.03 (0.66)	0.22 (0.001)
PBDE 47	0.05 (0.47)	0.16 (0.02)	0.19 (0.005)
PBDE 66	0.06 (0.38)	0.14 (0.04)	0.01 (0.86)
PBDE 85	0.09 (0.19)	0.18 (0.009)	0.10 (0.14)
PBDE 99	0.10 (0.14)	0.24 (0.0005)	0.14 (0.05)
PBDE 100	0.06 (0.36)	0.10 (0.14)	0.12 (0.08)
PBDE 153	0.06 (0.41)	−0.08 (0.23)	0.06 (0.39)

PBDE 154	0.08 (0.27)	0.20 (0.005)	0.12 (0.09)
PBDE 183	0.04 (0.58)	−0.01 (0.94)	0.14 (0.04)
Polychlorinated biphenyls (PCB)			
PCB 28	−0.03 (0.69)	0.07 (0.27)	−0.04 (0.53)
PCB 44	−0.03 (0.69)	0.07 (0.29)	−0.06 (0.42)
PCB 49	−0.03 (0.63)	0.06 (0.37)	−0.04 (0.56)
PCB 52	−0.03 (0.65)	0.07 (0.31)	−0.05 (0.47)
PCB 66	−0.02 (0.77)	0.08 (0.26)	−0.03 (0.69)
PCB 74	0.06 (0.36)	0.08 (0.27)	0.04 (0.52)
PCB 87	0.05 (0.47)	0.09 (0.17)	0.11 (0.13)
PCB 99	0.22 (0.001)	0.06 (0.36)	0.27 (0.0002)
PCB 101	0.02 (0.75)	0.08 (0.21)	0.03 (0.63)
PCB 105	0.20 (0.003)	0.11 (0.11)	0.33 (<0.0001)
PCB 110	0.04 (0.57)	0.07 (0.27)	0.01 (0.87)
PCB 114	0.16 (0.02)	−0.01 (0.89)	0.12 (0.10)
PCB 118	0.25 (0.0002)	0.07 (0.29)	0.31 (<0.0001)
PCB 128	0.04 (0.51)	0.10 (0.13)	0.16 (0.02)
PCB 138	0.30 (<0.0001)	0.00 (0.98)	0.25 (0.0006)
PCB 146	0.32 (<0.0001)	−0.04 (0.56)	0.20 (0.005)
PCB 149	0.17 (0.01)	0.09 (0.21)	0.09 (0.21)
PCB 151	0.16 (0.02)	0.06 (0.41)	0.08 (0.27)
PCB 153	0.33 (<0.0001)	−0.07 (0.33)	0.22 (0.002)
PCB 156	0.21 (0.002)	−0.14 (0.03)	0.09 (0.20)
PCB 157	0.22 (0.001)	−0.14 (0.04)	0.05 (0.44)
PCB 167	0.29 (<0.0001)	0.04 (0.52)	0.18 (0.01)
PCB 170	0.35 (<0.0001)	−0.10 (0.13)	0.20 (0.004)
PCB 172	0.33 (<0.0001)	0.01 (0.84)	0.19 (0.007)
PCB 177	0.33 (<0.0001)	0.02 (0.81)	0.24 (0.0005)
PCB 178	0.26 (0.0001)	−0.06 (0.37)	0.20 (0.005)
PCB 180	0.37 (<0.0001)	−0.12 (0.07)	0.18 (0.01)
PCB 183	0.35 (<0.0001)	0.07 (0.34)	0.27 (0.0002)
PCB 187	0.32 (<0.0001)	−0.01 (0.85)	0.23 (0.001)
PCB 189	0.18 (0.007)	−0.10 (0.16)	0.06 (0.35)
PCB 194	0.40 (<0.0001)	−0.11 (0.11)	0.16 (0.02)
PCB 195	0.28 (<0.0001)	−0.07 (0.29)	0.18 (0.01)
PCB 196	0.42 (<0.0001)	0.00 (0.98)	0.21 (0.003)
PCB 201	0.39 (<0.0001)	−0.06 (0.35)	0.15 (0.04)
PCB 206	0.41 (<0.0001)	−0.02 (0.79)	0.19 (0.008)
PCB 209	0.45 (<0.0001)	−0.11 (0.12)	0.18 (0.009)
Organochlorine pesticides			
HCB	0.16 (0.02)	0.02 (0.72)	0.44 (<0.0001)
β-HCH	0.12 (0.09)	−0.03 (0.70)	0.15 (0.03)
γ-HCH	0.03 (0.69)	0.00 (0.95)	−0.07 (0.34)
Oxychlordane	0.19 (0.004)	0.05 (0.43)	0.31 (<0.0001)
Trans-nonachlor	0.17 (0.01)	0.07 (0.27)	0.28 (<0.0001)
p,p′-DDT	0.13 (0.06)	0.00 (0.99)	0.15 (0.03)
o,p′-DDT	0.16 (0.02)	0.00 (1.00)	0.17 (0.01)
p,p′-DDE	0.28 (<0.0001)	−0.07 (0.33)	0.28 (<0.0001)
Mirex	0.15 (0.02)	0.01 (0.92)	0.06 (0.36)
Perfluoroalkyls and polyfluoroalkyls (PFAAs)			
Et-PFOSA-AcOH	0.17 (0.02)	−0.05 (0.46)	−0.06 (0.40)
Me-PFOSA-AcOH	0.00 (0.99)	−0.06 (0.39)	−0.06 (0.41)

PFDeA	0.14 (0.04)	−0.02 (0.74)	0.08 (0.27)
PFNA	0.10 (0.15)	0.02 (0.80)	0.12 (0.07)
PFOSA	−0.02 (0.77)	−0.04 (0.58)	−0.02 (0.72)
PFOS	0.10 (0.13)	−0.04 (0.56)	0.08 (0.26)
PFOA	−0.04 (0.58)	−0.04 (0.60)	0.12 (0.08)

Correlation coefficients from the multiple imputations were combined using Fisher's z transformation. Serum lipids were estimated from the Phillips 1989 equation and re-scaled and natural-log transformed. Values are Pearson correlation coefficients (*p*-value). *p*-values < 0.05 are bolded. Average limit of detection (LOD) across all participants (each sample has its own LOD).

3. Table S3

Table S3. Estimates from linear regression of the association between persistent organic pollutants and gestational weight gain stratified by pre-pregnancy body mass index (BMI) status [a].

Chemical or Congener	Total GWG		AUC	
	BMI < 25 kg/m² Beta (95% CI)	BMI ≥ 25 kg/m² Beta (95% CI)	BMI < 25 kg/m² Beta (95% CI)	BMI ≥ 25 kg/m² Beta (95% CI)
Polybrominated biphenyl (PBB)				
PBB 153	−0.42 (−1.27, 0.43)	−1.31 (−3.48, 0.87)	−175.29 (−421.41, 70.82)	−239.82 (−716.47, 236.82)
Polybrominated diphenyl ethers (PBDE)				
PBDE 17	−0.34 (−1.09, 0.41)	0.73 (−1.55, 3.01)	−126.74 (−344.60, 91.12)	127.81 (−360.84, 616.46)
PBDE 28	−0.20 (−1.06, 0.65)	0.09 (−2.59, 2.78)	−65.53 (−315.00, 183.94)	−476.64 (−1068.50, 115.23)
PBDE 47	−0.02 (−1.09, 1.04)	0.99 (−0.78, 2.76)	−25.23 (−334.83, 284.36)	−296.37 (−722.17, 129.43)
PBDE 66	−0.16 (−1.08, 0.76)	−1.21 (−2.89, 0.48)	−31.72 (−298.28, 234.83)	−376.04 (−734.05, −18.03)
PBDE 85	0.01 (−1.17, 1.19)	1.47 (−0.40, 3.34)	46.38 (−296.84, 389.60)	−236.86 (−708.60, 234.88)
PBDE 99	0.25 (−0.97, 1.47)	1.48 (−0.19, 3.14)	29.85 (−325.45, 385.14)	−164.69 (−593.44, 264.07)
PBDE 100	0.01 (−0.89, 0.92)	0.34 (−1.55, 2.23)	143.59 (−119.12, 406.30)	−357.41 (−775.26, 60.43)
PBDE 153	0.01 (−0.77, 0.78)	0.24 (−1.81, 2.28)	194.07 (−28.63, 416.77)	−142.36 (−586.73, 302.00)
PBDE 154	−0.18 (−1.30, 0.93)	1.35 (−0.39, 3.10)	40.23 (−282.86, 363.32)	−328.21 (−808.91, 152.50)
PBDE 183	−0.43 (−1.23, 0.38)	−0.10 (−2.02, 1.83)	−127.55 (−361.09, 105.98)	18.29 (−399.15, 435.74)
Polychlorinated biphenyls (PCB)				
PCB 28	4.36 (−14.91, 23.62)	−0.42 (−1.47, 0.63)	4044.90 (−1506.73, 9596.53)	−38.28 (−268.70, 192.14)
PCB 44	0.43 (−10.17, 11.04)	−0.37 (−1.43, 0.69)	112.69 (−2740.38, 2965.77)	−24.45 (−256.77, 207.87)
PCB 49	4.16 (−4.01, 12.34)	−0.41 (−1.47, 0.65)	1187.15 (−1125.50, 3499.80)	−37.66 (−269.72, 194.40)
PCB 52	8.77 (−0.85, 18.38)	−0.40 (−1.46, 0.65)	2029.51 (−853.53, 4912.55)	−32.67 (−263.92, 198.58)
PCB 66	7.87 (−1.82, 17.57)	−0.45 (−1.50, 0.61)	3097.98 (315.94, 5880.01)	−45.54 (−276.42, 185.33)
PCB 74	0.18 (−3.06, 3.42)	−0.37 (−1.45, 0.72)	225.98 (−712.50, 1164.46)	−36.59 (−273.42, 200.25)

PCB 87	1.24<br>(−0.11, 2.58)	−0.44<br>(−1.75, 0.87)	351.21<br>(−52.25, 754.67)	−66.98<br>(−353.40, 219.45)
PCB 99	0.23<br>(−0.95, 1.40)	−0.33<br>(−1.91, 1.24)	137.83<br>(−208.77, 484.43)	−89.89<br>(−433.62, 253.83)
PCB 101	2.15<br>(0.28, 4.03)	−0.94<br>(−2.48, 0.61)	461.11<br>(−131.46, 1053.67)	−118.18<br>(−456.41, 220.05)
PCB 105	0.28<br>(−0.94, 1.50)	−0.64<br>(−2.24, 0.95)	149.67<br>(−205.22, 504.57)	−173.77<br>(−521.23, 173.68)
PCB 110	0.25<br>(−0.81, 1.31)	−0.83<br>(−2.32, 0.67)	21.67<br>(−296.87, 340.21)	−107.30<br>(−436.43, 221.83)
PCB 114	0.10<br>(−0.75, 0.95)	0.00<br>(−1.91, 1.92)	94.28<br>(−151.86, 340.42)	−71.29<br>(−489.69, 347.12)
PCB 118	0.31<br>(−0.93, 1.54)	−0.39<br>(−1.99, 1.20)	194.62<br>(−162.72, 551.97)	−119.64<br>(−466.63, 227.35)
PCB 128	1.52<br>(−0.24, 3.29)	−0.51<br>(−2.41, 1.39)	454.69<br>(−391.42, 1300.80)	−223.47<br>(−635.66, 188.72)
PCB 138	−0.33<br>(−1.55, 0.89)	−0.11<br>(−1.87, 1.64)	−15.46<br>(−369.65, 338.72)	−24.20<br>(−407.37, 358.96)
PCB 146	−0.48<br>(−1.64, 0.69)	0.13<br>(−1.69, 1.95)	−35.67<br>(−377.51, 306.16)	−8.68<br>(−405.56, 388.20)
PCB 149	2.10<br>(−3.28, 7.47)	−5.54<br>(−11.68, 0.59)	993.13<br>(−888.84, 2875.09)	−914.96<br>(−2264.67, 434.76)
PCB 151	8.70<br>(0.52, 16.87)	−3.33<br>(−8.11, 1.45)	1619.97<br>(−2011.45, 5251.39)	−706.19<br>(−1750.69, 338.31)
PCB 153	−0.39<br>(−1.58, 0.80)	0.06<br>(−1.83, 1.94)	−46.41<br>(−392.78, 299.96)	0.58<br>(−411.34, 412.50)
PCB 156	−0.18<br>(−1.06, 0.70)	0.55<br>(−1.51, 2.62)	−62.61<br>(−317.08, 191.85)	31.73<br>(−415.95, 479.40)
PCB 157	−0.01<br>(−0.86, 0.84)	0.04<br>(−1.94, 2.01)	−21.71<br>(−268.34, 224.91)	19.68<br>(−410.59, 449.94)
PCB 167	0.84<br>(−0.25, 1.92)	0.18<br>(−1.47, 1.84)	311.93<br>(−1.24, 625.10)	6.03<br>(−355.79, 367.84)
PCB 170	−0.74<br>(−1.92, 0.44)	0.71<br>(−1.40, 2.82)	−197.72<br>(−541.26, 145.82)	147.75<br>(−310.78, 606.28)
PCB 172	−0.77<br>(−1.94, 0.40)	−0.11<br>(−1.92, 1.70)	−138.99<br>(−482.32, 204.35)	4.08<br>(−390.22, 398.38)
PCB 177	−0.86<br>(−3.13, 1.42)	0.19<br>(−2.56, 2.94)	−44.87<br>(−709.65, 619.92)	151.30<br>(−448.95, 751.56)
PCB 178	−0.48<br>(−1.72, 0.77)	0.51<br>(−1.53, 2.55)	−53.54<br>(−416.56, 309.49)	278.55<br>(−164.27, 721.36)
PCB 180	−0.66<br>(−1.84, 0.52)	0.81<br>(−1.21, 2.83)	−144.46<br>(−488.14, 199.22)	199.61<br>(−238.32, 637.54)
PCB 183	−0.29<br>(−2.18, 1.60)	−0.43<br>(−2.75, 1.88)	189.95<br>(−358.13, 738.03)	12.43<br>(−492.95, 517.80)
PCB 187	−0.86<br>(−2.42, 0.70)	0.22<br>(−1.92, 2.35)	−151.53<br>(−604.66, 301.60)	80.35<br>(−386.20, 546.91)
PCB 189	−0.49<br>(−1.37, 0.39)	0.22<br>(−1.61, 2.05)	−112.59<br>(−368.96, 143.78)	127.18<br>(−270.70, 525.07)
PCB 194	−0.36<br>(−1.41, 0.68)	1.33<br>(−0.49, 3.15)	−61.03<br>(−366.58, 244.52)	306.69<br>(−91.50, 704.87)
PCB 195	−0.44<br>(−1.62, 0.74)	0.55<br>(−1.26, 2.37)	−74.38<br>(−417.39, 268.64)	287.24<br>(−103.54, 678.03)
PCB 196	−0.51<br>(−1.79, 0.76)	0.98<br>(−0.86, 2.83)	−38.04<br>(−409.73, 333.66)	223.19<br>(−180.33, 626.72)
PCB 201	−0.57<br>(−1.87, 0.73)	1.10<br>(−0.77, 2.96)	3.72<br>(−375.03, 382.47)	254.14<br>(−155.78, 664.05)
PCB 206	−0.83<br>(−2.01, 0.35)	0.95<br>(−0.69, 2.59)	−55.59<br>(−400.73, 289.55)	271.22<br>(−86.20, 628.65)

PCB 209	−0.74<br>(−1.78, 0.29)	0.87<br>(−0.61, 2.36)	−37.65<br>(−339.94, 264.65)	257.88<br>(−63.42, 579.18)
Organochlorine pesticides				
HCB	**1.09**<br>**(0.06, 2.11)**	−0.10<br>(−1.90, 1.70)	108.29<br>(−213.01, 429.60)	−176.09<br>(−564.78, 212.60)
β-HCH	0.65<br>(−0.02, 1.32)	−0.23<br>(−6.83, 6.37)	−249.66<br>(−746.53, 247.21)	457.86<br>(−953.48, 1869.20)
γ-HCH	0.49<br>(−0.51, 1.49)	0.03<br>(−1.82, 1.87)	71.00<br>(−220.26, 362.26)	−10.56<br>(−409.74, 388.61)
Oxychlordane	−1.00<br>(−2.02, 0.02)	−0.18<br>(−1.93, 1.56)	−226.72<br>(−526.46, 73.01)	−14.40<br>(−393.22, 364.42)
Trans-nonachlor	−0.79<br>(−1.89, 0.32)	−0.24<br>(−1.79, 1.30)	−212.15<br>(−535.46, 111.16)	69.57<br>(−267.74, 406.88)
p,p′-DDT	0.45<br>(−0.22, 1.11)	1.38<br>(−3.19, 5.96)	−327.73<br>(−667.66, 12.19)	−542.53<br>(−1599.91, 514.84)
o,p′-DDT	0.58<br>(−0.15, 1.32)	0.79<br>(−2.08, 3.67)	−128.97<br>(−492.90, 234.96)	−211.15<br>(−829.09, 406.78)
p,p′-DDE	0.55<br>(−0.32, 1.41)	−0.29<br>(−2.22, 1.64)	−62.70<br>(−380.31, 254.92)	1.25<br>(−418.29, 420.79)
Mirex	0.28<br>(−1.47, 2.04)	0.33<br>(−2.34, 3.01)	326.64<br>(−180.35, 833.62)	267.54<br>(−311.15, 846.23)
Perfluoroalkyls and polyfluoroalkyls (PFAAs)				
Et-PFOSA-AcOH	−0.64<br>(−1.54, 0.26)	0.12<br>(−1.79, 2.04)	−44.51<br>(−310.19, 221.16)	58.73<br>(−358.67, 476.14)
Me-PFOSA-AcOH	−0.72<br>(−1.69, 0.26)	0.11<br>(−1.42, 1.64)	−144.77<br>(−430.83, 141.29)	−39.56<br>(−375.94, 296.82)
PFDeA	0.36<br>(−0.64, 1.36)	−0.33<br>(−1.84, 1.17)	184.06<br>(−110.50, 478.63)	−47.11<br>(−376.77, 282.55)
PFNA	0.62<br>(−0.30, 1.54)	−1.09<br>(−2.97, 0.80)	253.07<br>(−12.67, 518.81)	−180.69<br>(−594.86, 233.47)
PFOSA	0.39<br>(−0.62, 1.41)	0.77<br>(−0.75, 2.28)	28.67<br>(−267.40, 324.73)	193.06<br>(−136.67, 522.79)
PFOS	0.72<br>(−0.20, 1.63)	−0.31<br>(−2.01, 1.39)	280.29<br>(13.71, 546.86)	56.99<br>(−328.36, 442.34)
PFOA	0.31<br>(−0.60, 1.21)	−0.22<br>(−1.98, 1.54)	176.69<br>(−86.09, 439.46)	−191.81<br>(−575.09, 191.46)

AUC: Area under the curve; CI: Confidence interval; GWG: Gestational weight gain; OR: Odds ratio.
[a] Values are beta (95% CI) estimated from multivariable linear regression. Adjusted for pre-pregnancy non-fasting serum lipids.

© 2016 by the authors; licensee MDPI, Basel, Switzerland. This article is an open access article distributed under the terms and conditions of the Creative Commons by Attribution (CC-BY) license (http://creativecommons.org/licenses/by/4.0/).
